# Symmetry perception with spiking neural networks

**DOI:** 10.1038/s41598-021-85232-3

**Published:** 2021-03-11

**Authors:** Jonathan K. George, Cesare Soci, Mario Miscuglio, Volker J. Sorger

**Affiliations:** 1 Department of Electrical and Computer Engineering, George Washington University, Washington, DC USA; 2 School of Physical and Mathematical Sciences and School Electrical and Electronic Engineering, Nanyang Technological University, Singapore, Singapore

**Keywords:** Electrical and electronic engineering, Information technology

## Abstract

Mirror symmetry is an abundant feature in both nature and technology. Its successful detection is critical for perception procedures based on visual stimuli and requires organizational processes. Neuromorphic computing, utilizing brain-mimicked networks, could be a technology-solution providing such perceptual organization functionality, and furthermore has made tremendous advances in computing efficiency by applying a spiking model of information. Spiking models inherently maximize efficiency in noisy environments by placing the energy of the signal in a minimal time. However, many neuromorphic computing models ignore time delay between nodes, choosing instead to approximate connections between neurons as instantaneous weighting. With this assumption, many complex time interactions of spiking neurons are lost. Here, we show that the coincidence detection property of a spiking-based feed-forward neural network enables mirror symmetry. Testing this algorithm exemplary on geospatial satellite image data sets reveals how symmetry density enables automated recognition of man-made structures over vegetation. We further demonstrate that the addition of noise improves feature detectability of an image through coincidence point generation. The ability to obtain mirror symmetry from spiking neural networks can be a powerful tool for applications in image-based rendering, computer graphics, robotics, photo interpretation, image retrieval, video analysis and annotation, multi-media and may help accelerating the brain-machine interconnection. More importantly it enables a technology pathway in bridging the gap between the low-level incoming sensor stimuli and high-level interpretation of these inputs as recognized objects and scenes in the world.

## Introduction

In neuromorphic computing the architecture of the biological brain is applied to artificial computers to both increase the efficiency of computing and to mimic the intelligence of the human mind. While artificial neural networks have become increasingly complex, they have largely modeled the connections between neurons as synaptic weighting and summing through a nonlinear activation function. However, biological neurons exist physically in a three-dimensional space implying that information in the brain is, in its most basic form, distributed in at least three dimensions. Furthermore, communication between neurons is not instantaneous and depends not only on the distance in the three-dimensional space but on the density and type of connections, including neurophil, axons and dendrites, between them, creating a rich N-dimensional time dependent space in addition to the traditional N-dimensional weight dependent space. Here, we show how these simple time interactions applied to an elementary spiking model of the neuron can result in complex outcomes, such as the identification of mirror symmetry, extending our conference presentation^1^.

The ability to rapidly identify symmetry and anti-symmetry is an essential attribute of intelligence. Symmetry perception is a central process in human vision and may play a key role in human 3D visualization^2^. While previous work in understanding neuron symmetry perception has concentrated on the neuron as an integrator^3^ and midpoint linearity^4^, here we show how the coincidence detecting property of the spiking neuron can be used to reveal symmetry density in spatial data. We develop a method for synchronizing symmetry-identifying spiking artificial neural networks to enable layering and feedback in the network, to form a recurrent neural network (RNN). We show a method for building a network capable of identifying symmetry density between sets of data and present a digital logic implementation demonstrating an $8\times 8$ leaky-integrate-and-fire symmetry detector in a field-programmable gate array. Our results show that the efficiency of spiking neural networks can be harnessed to rapidly identify symmetry in spatial data with applications in image processing, 3D computer vision, and robotics.

The human visual system is able to detect mirror symmetry rapidly^5^. Furthermore, symmetry detection in human vision is being hypothesized to be essential for 3D visualization^6^. Symmetry preference is also found in insects and birds^7,8^. These examples point to a neurological origin of symmetry detection. While line integration has been proposed as a method for symmetry perception in human vision^9^ and in spiking neural networks^3^, a more fundamental role may be played by neural coincidence detection; in biology the spiking neural networks of the brain have been shown to be capable of both integration and coincidence detection^10,11^. These spiking neurons have been observed to fire in a time-dependent manner forming strongly connected clusters^12^ known as polychronous neural groups (PNGs)^9^. Both pattern recognition and computing have been achieved in artificial neural networks with polychronous behavior^13^. However, the connection between symmetry and coincident spiking polychronous neural networks has not been explored, nor has symmetry detection using only single layer delay in spiking artificial neural networks been demonstrated. While many algorithms for finding symmetry in images and feature sets of images have been developed^14–19^, it is difficult to translate them to run on power-efficient neuromorphic hardware platforms^20,21^. Here we present a formal definition of geometric symmetry as the amplitude of a tensor space of the distribution of distance. We then show how a specific configuration of a spiking neural network can act on its inputs in a manner identical to a threshold applied to the tensor symmetry space, firing at the points of high mirror symmetry. As an example, we demonstrate a network both in software and in a Field Programmable Gate Array (FPGA) and validate symmetry-recognition capability of an artificial spiking neural network on a) geospatial data sets demonstrating automated dwelling detection and b) on shapes of military aircraft where mirror symmetry enables fingerprint-like rapid detection such as for automatic target recognition (ATR). The symmetry-associating behavior of spiking neural networks has immediate applications in image processing and is consistent with our intuition that the ability to identify symmetry is indeed supported by artificial neural intelligence.

### Symmetry density

Symmetry can be broadly defined as a self-similarity in logic or a dataset. Geometric symmetries are self-similarities in a spatial dataset over a transformation. The transformation can consist of 1) mirror symmetry across an axis, 2) scaling symmetry where the transformation changes the size of some portion of the data, 3) rotational symmetry where some portion is of the data is rotated, 4) skew symmetry where the data is symmetric across a skew transformation, or 5) any combination of affine transformations. In this work, our focus is on geometric mirror symmetry which has been hypothesized to play an important role in human visual processing^6^.

Spatial self-similarity implies a transformation of some subset(s) of the data in a space will result again in the same subset of data. If the transform is folding the space, then both subsets of data will be equidistant to the folding line. In detecting these mirror symmetries, our problem becomes one of detecting peaks in the equidistant distribution of data.

#### Symmetry density in spiking neural networks

Spiking network enables coincidence detection, and coincidence detection enables mirror symmetry density detection. A spiking neural network is a type of artificial neural network that models the spiking observed in the neuron cells of the brain, in this sense it is Neuromorphic, taking on the form of a neuron. By concentrating its energy into a short time span the spike is an efficient encoding method in a noisy environment^12,13,22^.

A simple type of spiking neural network is the Leaky Integrate and Fire (LIF) model^13,23^, where each neuron integrates its inputs in time while simultaneously leaking from the accumulator. When the accumulator passes a threshold level it fires, generating a signal spike. The leak creates a temporal dependence on the past, thus adding memory to the neuron. The LIF model can be formulated mathematically^13,23^ as

1 $$\begin{aligned} u\left( t\right) =RI\left( t\right) -\tau _m\frac{du}{dt} \end{aligned}$$

where the voltage *u* is a function of current with a leaky term $\tau _m\frac{du}{dt}$ that depends on the change in voltage with time, and $\tau _m$ is the relaxation time constant of the signal leak to reach threshold.

LIF neurons have been realized in analog electronics^24,25^, in digital electronics with event-driven processors^26,27^, and in photonics with optical nonlinear materials^28^. A simple method for building a digital event-driven system with delay in a FPGA or application-specific integrated circuit (ASIC), is to approximate the propagation of spikes in digital form as bits moving through long shift registers. A 1 bit is placed at the input of the shift register to represent an incoming spike and in each clock cycle the bits in the register are shifted by one position until they arrive at the output where they are summed into a separate leaky register. If the leaky accumulation register reaches a threshold, 1 bits are placed at all of the neuron’s output shift registers. While this method potentially requires more resources than the alternative of using event counters, it intuitively maps to the role of spikes moving in the axon of a neuron.

**Figure 1:**
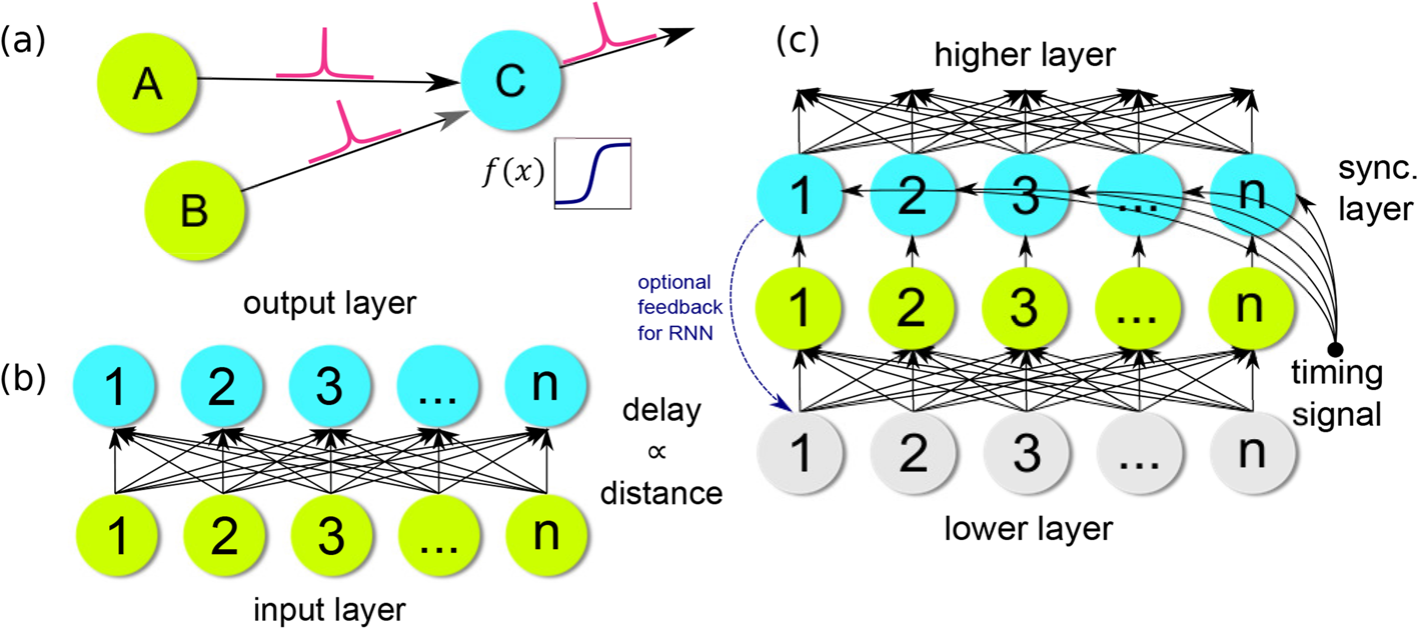
When neuron A and neuron B fire simultaneously, and if both neurons are equidistant from neuron C, then the pulse propagating from A to C will arrive at C at the same time as the pulse propagating from B to C (**a**). The threshold at C is set such that it is higher than the amplitude of either individual pulse. Neuron C will fire if, and only if, both pulses arrive time-synchronized. In this way neuron C acts as a coincidence detector. Each input layer neuron is connected to every output layer neuron with a delay proportional to the distance between the input and output points in Euclidean space (**b**). If all activated input neurons fire simultaneously, and only one pulse is allowed per cycle, the output node with highest symmetry will be the output node with the greatest number of pulses arriving simultaneously. To detect hierarchical geometric symmetry a synchronization layer is added above the original output layer (**c**). The synchronization layer has a slower integration time than the original output layer, storing the pulses until a timing signal releases them to the next higher layer simultaneously. In recurrent variations of the network (e.g. RNN, recurrent neural network), feedback from the output of the synchronization layer is connected back to a lower level input layer. See text for details of operation.

To understand how spatial symmetry affects the result of a LIF neural network, consider a simple neural network consisting of two input neurons A and B connected to a single output neuron C (Fig. 1). This three-neuron system acts as a coincidence detector. A spike propagating from A to C arrives at C after some time $\mathrm {\Delta }t_A$. Similarly, a spike propagating from B to C arrives at C after some time $\mathrm {\Delta }t_B$. If neuron C is assigned a threshold that is just higher than the individual spikes output from either A or B, then the neuron C will only fire when both spikes arrive at C before the leaky time constant $\tau _m$ reduces the power below threshold at C. In this configuration, C is a temporal coincidence detector.

**Figure 2:**
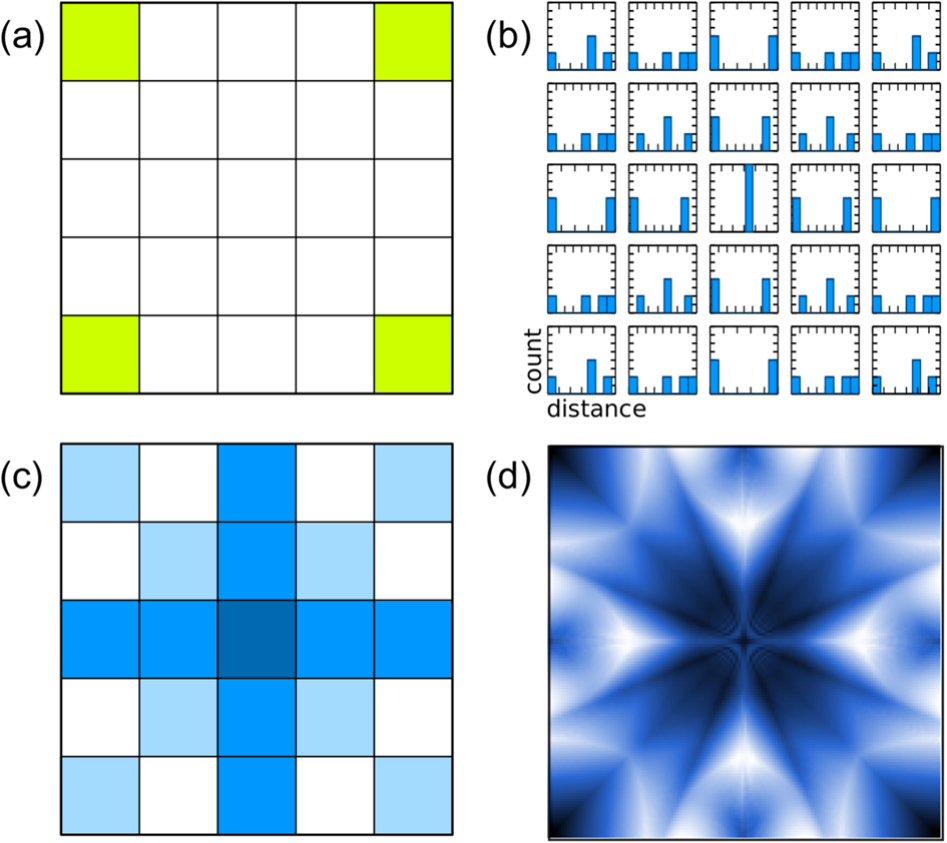
The algorithm transforms the input data points (the four corners of a Cartesian space in this example) (**a**) into an array of histograms of distances (**b**) where each bin counts the distance from the point in space to each data point. The peaks of the set of histograms are used to create a density of symmetry (**c**) overlaying the original space. Applying the algorithm repeatedly with feedback in a high-resolution space produces a fractal-like pattern (**d**) of hierarchical symmetry.

To obtain symmetry detection from coincidence detection, assume a constant propagation speed across the system, where propagation time is proportional to distance, and synchronous firing of input neuron A and B. Now, the spike arrival times $\mathrm {\Delta }t_A$ and $\mathrm {\Delta }t_B$ will be dependent only on propagation distances $d_A$ and $d_B$. In an $N \times N$ connected network, the arrival times will be distributed proportionally with distance, closer pulses arriving first and farther pulses arriving later. This is equivalent to the tensor space *D* of our original definition (Fig. 2).

If the two distances are equal the propagation times will be equal and the two spikes will arrive together at C, pushing it over the threshold and causing it to fire. On the other hand, if the distances are not equal, the propagation times will be different, the two spikes will arrive separately at C and the neuron will not fire. In this way, synchronized neurons in spiking neural networks act as the distance-relating components in the distribution elements of our tensor space *D*, and the threshold at neuron C acts as the second half of our symmetry algorithm, selecting the peaks out of the distance distributions in *D*. This method effectively, enables the neuron to act as a coincidence detector (Fig. 1).

Extending upon the three-neuron system, this concept of coincidence detection via delay can be brought to the entire input space (Fig. 1b). Each neuron of the input layer is connected to each neuron of the output layer ($N \times N$ connectivity) with a delay proportional to the distance between the two points in the space. For example, in a Cartesian space if input neuron 1 is point x = 0, y = 0, and output neuron 3 is the point x = 5, y = 4, the delay would be proportional to $\sqrt{25+16}\approx 6.4$. As stated before, the coincidence detecting property of the spiking neuron is selecting equidistant points.

While here we realize the algorithm in a GPU and FPGA, other neuron LIF architectures exist and it is of interest to define the bounds in which any architecture must operate. The speed (i.e. delay) of such a system in the ultimate physical limit is defined by the sum of the input layer firing delay, the pulse propagation delay, and the delay of the threshold layer. Assuming speed of light for propagation delay, the resolution of the device is set by its ability to distinguish individual pulses within the time of propagation. The worst case is the time delay of the smallest distance, given the shortest distance between individual data points in the space, i.e. the pitch of the array. Then the threshold layer must switch with at least $\mathrm {\Delta t}=d_p/c$ where $d_p$ is the pitch distance. From this we can state a lower bound for the average energy consumption of the threshold layer in the ultimate speed limit^29^.

2 $$\begin{aligned} E\ge \frac{\pi \hbar }{2\mathrm {\Delta t}}=\frac{\pi \hbar c}{2d_p} \end{aligned}$$

#### Definition of mirror symmetry density

We define mirror symmetry density as a continuous scalar field of the peaks of the tensor *D*. These peaks represent positions of strong equidistance within the space of our dataset *S*(*x*, *y*) . This is similar to definitions found in^10–12,14,19^, except that in those cases the problem is posed as a maximization of equidistant density on a histogram rather than simple density in^11^ and a minimization of transform energy in^10^. While both perspectives of the problem result in the same solution (the equidistant point will be halfway between two points of the image and will have the minimum transform energy), viewing the problem in terms of maximization allows us to map the problem into a spiking neural network, where thresholds are defined in terms of peak amplitude. For a space to have symmetry density it must have some definition of distance. This distance function f, or metric, between two points (A and B) must satisfy three conditions; First, it must be positive and equal for the same point.

3 $$\begin{aligned} f\left( a,b\right) \ge 0 \end{aligned}$$

Second, it must be coordinate-symmetric, i.e. adhering to coordinate-reversal symmetry

4 $$\begin{aligned} f\left( a,b\right) =f\left( b,a\right) \end{aligned}$$

Finally, it must satisfy the triangle inequality,

5 $$\begin{aligned} f\left( a,c\right) \le f\left( a,b\right) +\ f\left( b,c\right) \end{aligned}$$

which translates into the distance between two points being the shortest path. Meeting these criteria, a function is a metric. A common metric in the Euclidean space, for example, is the definition of distance in two-dimensional Cartesian coordinates:

6 $$\begin{aligned} \left\| B-A\right\|^{2} = \left( B_x - A_x\right) ^{2} + \left( B_y - A_y\right) ^{2} \end{aligned}$$

With this definition of distance we define the set of symmetry points, $S=\left\{ S_1,\ S_2,\ \ldots ,S_n\right\}$, as the point equidistant between two points A and B:

7 $$\begin{aligned} \exists X_A,X_B\ s.t.\ \left\| X_A-S_i\right\| =\left\| X_B-S_i\right\| \ \forall \ S_i \in S,\ X_A \in X,\ X_B \in X,\ X_A \ne X_B \end{aligned}$$

For the two points A and B, equation (7) defines the set of points, $S_i$ forming a line equidistant between the two points A and B. For, three points A, B, and C, there are three lines of symmetry formed by each of the pairs (A, B), (B, C), and (A, C) as well as possibly a single point equidistant to all three points. As the number of input points increases, the number of symmetry lines increases with the number of unique pairs of input points.

#### Algorithm

To understand the relationship of spiking neural networks with coincidence detection and to compare an artificial spiking neural network approach to a computational approach, it is useful to introduce an algorithmic method of ranking mirror symmetry.

**Figure 3:**
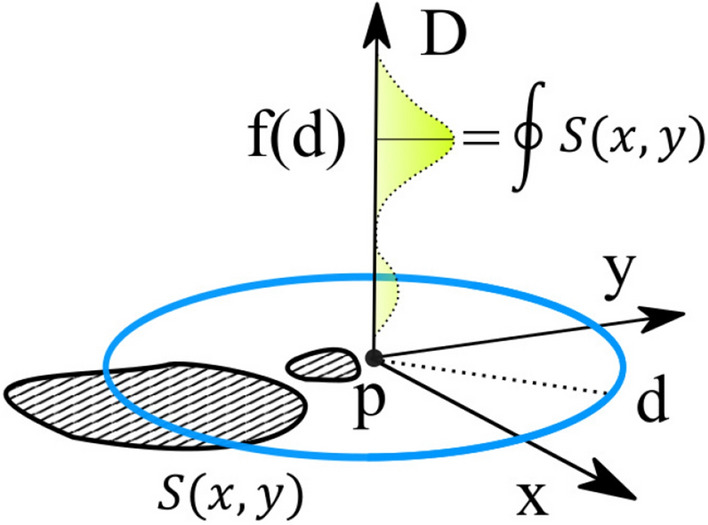
A tensor space *D* describes the distribution of distances at every point *p* to the image data surface *S*(*x*, *y*) , where the value at each position of the tensor $D_d$ corresponds to a distance *d* from point *p* and contains the contour integral of the surface *S*(*x*, *y*) around the circle at distance *d* from the point. In this way the tensor space *D* represents the density function of distances at all points in the surface to all other points in the surface. Equidistant points in the surface *S*(*x*, *y*) will generate peaks in the distribution along D and correspond to a symmetry density.

We define a tensor space *D* where at every point *p* in the space of our dataset *S*(*x*, *y*) the tensor describes the distribution of distance to every point in our dataset (Figs. 2, 3). Each dimension *d* along the tensor at point *p* is defined as the contour integral of the dataset *S*(*x*, *y*) about a circle of radius *d* centered at *p*. High symmetry points will correspond to peaks in the distribution of distances. We define Algorithm 1 to generate a discrete representation of this field.

With this algorithm symmetry points above a predefined threshold in the space can be identified. In $O(m*n)$ where m is the number of points in our space S, and n the number points in the set of input points in N. We note, that this algorithm can be applied iteratively where symmetries between input points (Fig. 2a) and other symmetry points create a hierarchy of symmetry points (Fig. 2d). The expected symmetry for the four inputs (green areas, Fig. 2a) result in the expected (1st order) symmetry shown in Fig. 2c. 
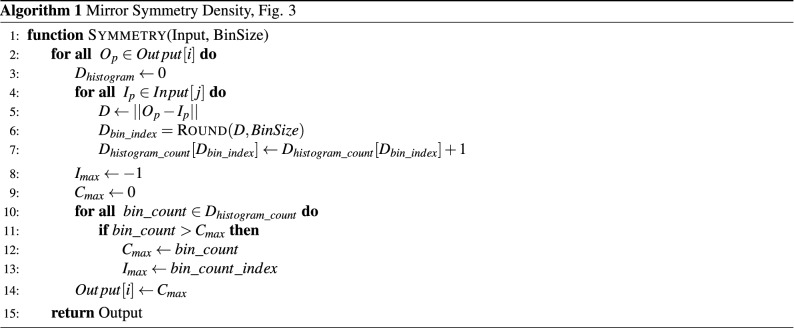

### Layering

Deep Neural Networks (DNNs) are inherently multilayered. The multilayered architecture gives DNNs the potential to create higher levels of abstraction than single layer networks. It is reasonable to ask if the geometric-symmetry identifying neural networks discussed here are able to support layering to similarly produce hierarchical levels of symmetry detection.

If we naively begin with the network of Fig. 1b and add a third layer on top of the original output layer, we quickly discover that the layering cannot be supported with the network as previously presented. While the pattern of symmetry will appear at the middle layer, each coincident set of pulses will arrive at different times. In order for geometric symmetry to be identified by coincidence detection in the top layer, input pulses (i.e. output from the middle layer) must either all start at the same time or, if initiated with time differences, these individual delays must be proportionally shorter than the respective connection delays. If the time differences were constant we could remove delay from the connection delay between the middle layer and the new output layer. However, the time differences are not predictable and are dependent on the symmetry in the data from the input layer. If we are to detect geometric symmetry in a multilayered network, we must adjust the network to account for the varying arrival times.

The LIF spiking neuron can act as a memory for synchronization, much like a register in digital logic. To see this behavior we imagine a set of pulses independently arriving at a set of slow leaking LIF neurons such that some of the neurons receive a pulse and some do not. The pulses may represent the 1s and 0s of the bits to be stored in the memory. Now, with the proper choice of threshold, the bit will be stored until either the neuron leaks away all of the energy received by the pulse or another pulse arrives, pushing the neuron over the threshold. If this second set, the pulses arrive at every neuron at the same time, the neurons will act as a synchronization stage, collecting pulses from their input and waiting until activated to simultaneously release the stored pulses to their outputs.

We can apply this concept of synchronization to the multilayered geometric symmetry-identifying network by adding a synchronization layer above the original output layer (Fig. 1c). This synchronization layer will have a slower leak rate than the layer below it so that it stores the symmetry points generated by the geometric symmetry detecting network below and, when a timing pulse arrives, release them at once to the symmetry detecting network above. In this way we can build a network to detect hierarchical geometric symmetry, i.e. the symmetry points of symmetry points.

Using the same process of synchronization we can also feed the output layer back to the input layer to create an RNN. The feedback allows the network to act on the symmetry points as well as the original data. The feedback output will differ from the multilayered network, as now original data will be compared to the generated symmetry points, whereas in the multilayered network each layer only computes the geometric symmetry of the layer below it. This feedback will create a dynamic system with results similar to repeated application of the symmetry algorithm presented earlier (Fig. 2d).

#### Sets

**Figure 4:**
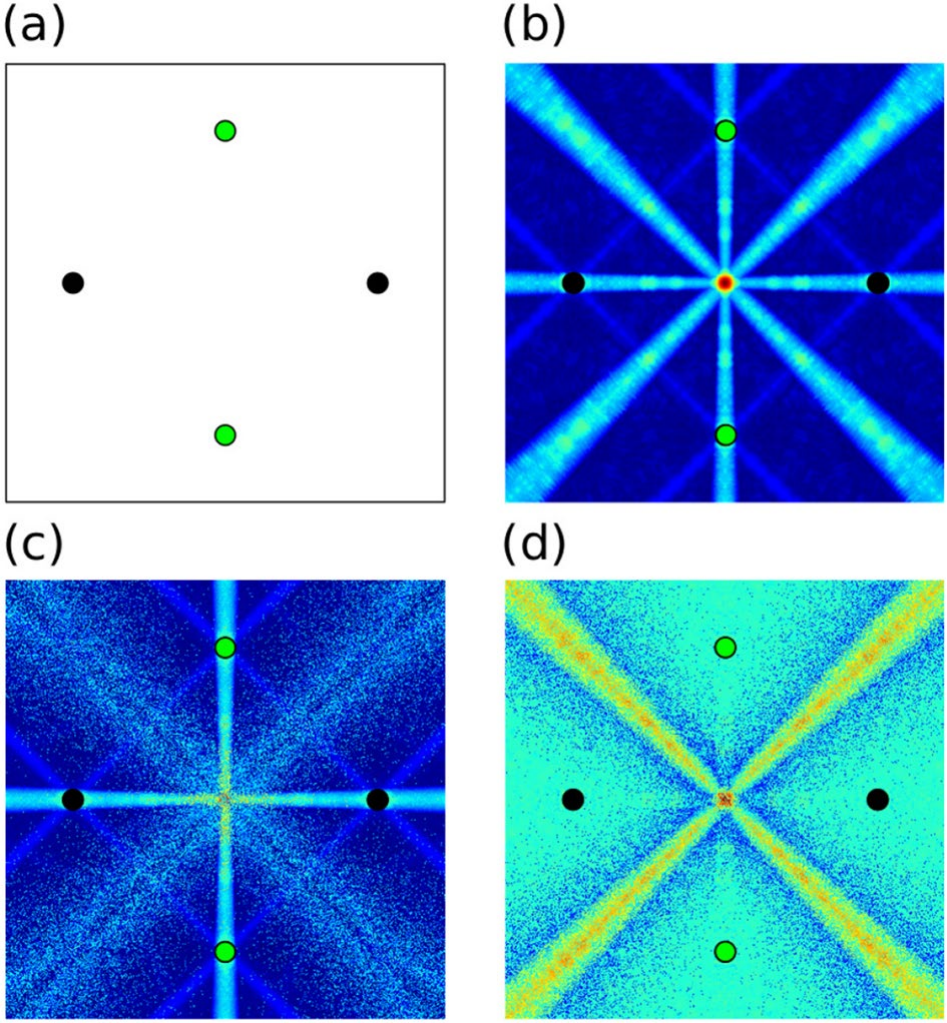
Two sets of data, green and black, (**a**) are processed by combining the inputs (**b**), resulting in both inter-set and intra-set symmetry at the output. To obtain a result with only inter-set symmetry, two distinct pseudorandom codes are applied to the two data sets, dispersing inter-set results (**c**). To obtain a result with only intra-set symmetry, the inter-set output (**c**) is subtracted from the original, or generated with inhibitory spiking in a spiking neural network, (**b**) to obtain the intra-set only result (**d**).

One of the primary applications of artificial neural networks is classification. Here the neural network decides the class of new input data based on prior training. In classification problems, it is often useful to compare sets of data. It may be interesting in this context to find the symmetry density within two different sets of data, intra-set symmetry, or between two different sets of data, inter-set symmetry. If we attempt to add two sets of data at the input layer (Fig. 4a), one for class A and one for class B, we will create a network that finds geometric symmetry between data in A and B but it will also find geometric symmetry from points in A to points in A and from points in B to points in B (Fig. 4b). To create a network that only detects inter-set or intra-set symmetry in the two sets of data we must amend the aforementioned architecture, as discussed next.

To detect intra-set symmetry two pseudorandom codes are generated, one for set A and one for set B. Elements of each pseudorandom code are associated with output connections of the network, N $\times$ N, that is one pseudorandom value is chosen for each distance value in the norm. When building delays for inputs from the space of set A, the pseudorandom code for A at each associated output connection is added to the delay. Similarly, for the space of set B delays are added from the pseudorandom code for set B. Each connection which previously had a delay directly proportional to distance is now perturbed by a small pseudorandom amount. This effectively re-codes the norm space in terms of the pseudorandom code, where before it was coded directly proportional to distance. The coincidence detectors at the output before correlated spikes of equal delay which corresponded to inputs of equal distance, now the coincidence detectors are still correlating spikes of equal but with perturbed delay which, again, corresponds to inputs of equal distance. Spikes originating within set A will have the same pseudorandom delays applied and will continue to be coincident. At the same time the delays between set A and set B will be randomized and the coincidence will be dispersed (Fig. 4c).

Similarly, to detect inter-set symmetry a second network is generated with connections from both sets of inputs. This results in a union of both intra-set and inter-set symmetry (Fig. 4b). At the output of this network inhibitory connections are applied from the output of the intra-set network. This results in a reduction in spiking from the intra-set network, effectively subtracting the intra-set result from the union to create the inter-set result (Fig. 4d).

These two techniques allow both types of set comparisons using only coincidence detection, delay, and spike inhibition. The results can be cascaded hierarchically, as discussed previously to perform complex comparisons between many sets of data.

#### Metrics

The concept of symmetry density is not limited to Cartesian coordinates or Euclidean distance. Other measures of distance can form varying types of equidistant symmetry. In the implementation of the algorithm in software, the Manhattan distance may be appealing as it can simplify the calculation of distance by eliminating the squares and square root of the Euclidean distance formula. Multiplication is at best ${\mathcal {O}}\left( n \log n \cdot 2^{\log ^*n}\right)$^30^ and square root is ${\mathcal {O}}\left( \log {n} F\left( n\right) \right)$ with Newton’s method, while addition and subtraction are ${\mathcal {O}}\left( \log N \right)$^31^.

However, this simplification has dramatic effects on the distribution of symmetry. In Euclidean space the equidistant point C is found by moving B circularly around point A, resulting in a single symmetry point for a circle. In Manhattan space horizontal and vertical positions are also equidistant having the effect of emphasizing horizontal, vertical, and diagonal lines in Manhattan space.

#### Spiking noise

To withstand noise, implementations of the symmetry density algorithm will need to set a threshold to determine which points are included in the dataset or weight the distribution with the value of the pixels. In the threshold case, the data point is either included or excluded from the distribution of distances based on whether the value of the pixel passes some threshold value. In a spiking neural network this is the activation threshold of the neuron receiving the input signal. The threshold can be set to the mean of the peak value across the space or some percentage of the mean to increase or decrease contrast.

In the weighting case the average value, or minimum value of the two equidistant pixels is applied as a weight to the value of the symmetry density in the same way that mass is applied as a weight in integration to find the center of mass of an object. This result can also be seen in long averages of a spiking neural network, where the repetition of spikes is proportional to the amplitude of the input signal.

Adding noise to the input causes the addition of noise in the distance distribution, raising the noise floor of the distribution. This noise decreases the separation between the peak of the distribution and the noise floor. Once the original peak is no longer detectable in the distribution, the output point in the symmetry density will become incorrect. The strength of noise where this occurs is dependent on the Signal to Noise Ratio (SNR) of the original symmetry density.

Gaussian noise is independent of the signal and will thus have a flat distance distribution. Adding Gaussian noise to the input will have a proportional effect on the SNR of the symmetry density. Any addition of noise in dB can simply be subtracted from the SNR of the original symmetry density to determine the resulting SNR (see further below, Fig. 8).

## Results

We hypothesize that the symmetric nature of human made objects would generate a higher level of symmetry density than natural objects. To test this hypothesis we created an experiment using the open source SpaceNet^32^ dataset. The SpaceNet dataset includes Are of Interest 1 (AOI 1), Rio de Janeiro which is a collection 7186 images of 50 cm imagery from DigitalGlobe’s WorldView-2 satellite of a mixture of farmland and buildings in Rio de Janeiro, Brazil. The dataset includes metadata for each of the 7186 tiles containing polygon outlines of buildings.

### Main experiment

Our experiment consisted of the following steps for each image in the dataset: 1. convert image from tiff to jpeg. 2. load first channel from jpeg 3. run Sobel-Feldman operator^33^ for edge detection 4. run symmetry function on GPU (Algorithm 3) on edge filter results with a threshold = 128, d = 50 px, and n = 177828. 5. save the result.

**Figure 5:**
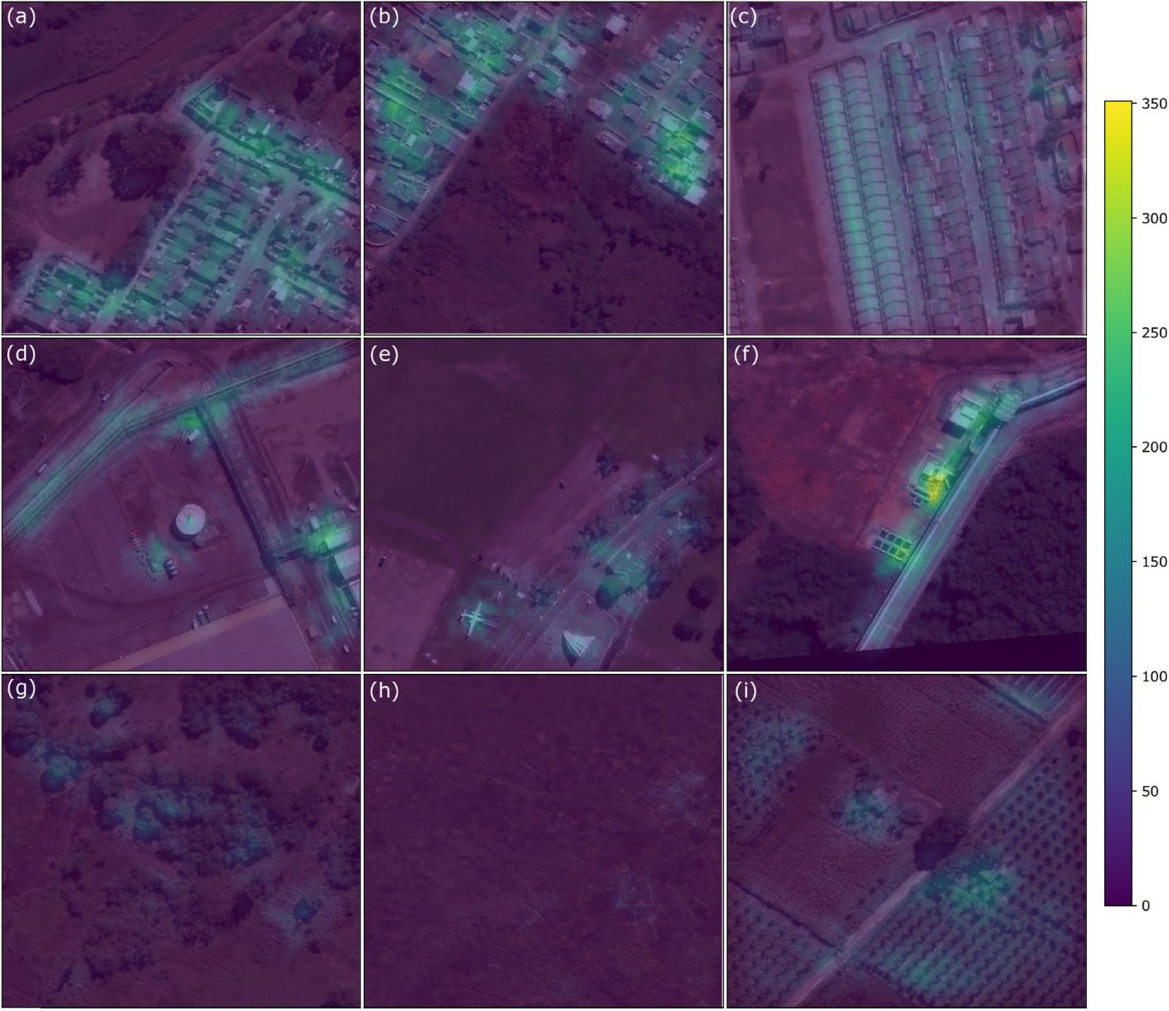
Samples from the experiment results with symmetry density overlayed on top of the original images on the same color scale (0 to 350), (**a**) image 1361 shows city structures with high symmetry density in the bottom-right and vegetation with low symmetry density in the upper-left portion, (**b**) image 1284 also showing symmetric urban areas in the upper-left and upper-right side, (**c**) image 5864 with rows of buildings with symmetric centers, (**d**) image 581 industrial center with circularly symmetric facility, (**e**) image 1014 oddly shaped building and potentially an aircraft in the lower left, (**f**) image 12 an industrial facility with highly symmetric tanks, (**g**) image 1363 show some symmetry density in the distribution of trees, (**h**) image 1395 show no symmetry density in an open field, and (**i**) image 1308 showing symmetric vegetation in fields.

**Figure 6:**
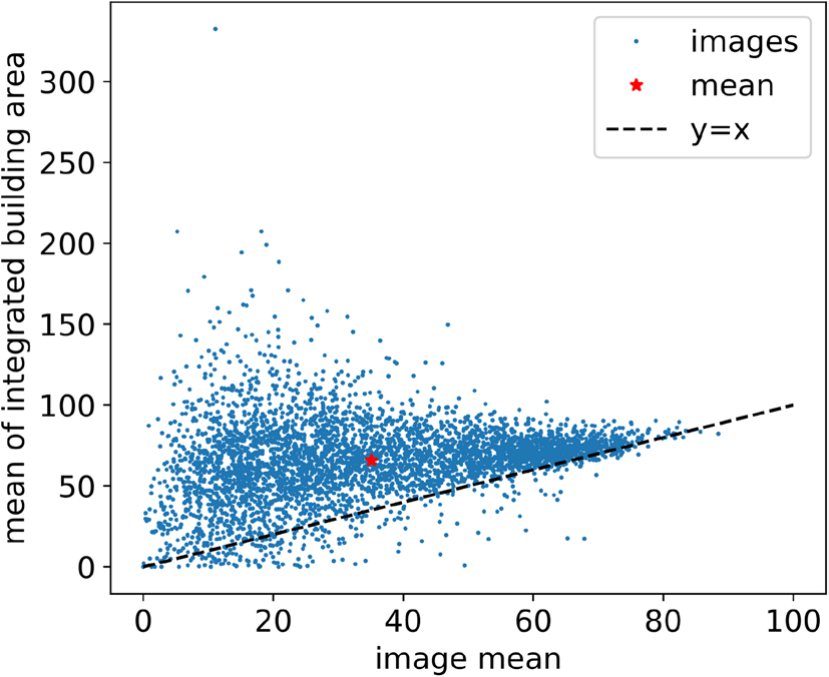
The mean of the symmetry density integrated within all building areas for each image containing buildings plotted against the mean of the symmetry density in the entire image shows that the area within buildings has a higher symmetry density than the average of the image for more than 93% of the images.

Samples of the result of the algorithm (Fig. 5) demonstrate the operating principal of the hypothesis with human made structures being highlighted more than natural vegetation. This can be empirically seen when plotting the total number of buildings in each tile vs the total symmetry density of the tile (Fig. 6). However, some types of human distributed vegetation such as crops are highly symmetric resulting in false positives.

### Scaling and noise

Pairs of points equidistant to a line of symmetry contribute to the signal along the line of symmetry while pairs of points not equidistant to a line of symmetry contribute to the noise along that line of symmetry. If there are multiple lines of symmetry in different directions and at different scales, the signal of any single line of symmetry will be reduced by the increased noise generated by the other lines of symmetry. In addition, if some portions of the data are not at all symmetric, they will further increase the noise along all lines of symmetry.

**Figure 7:**
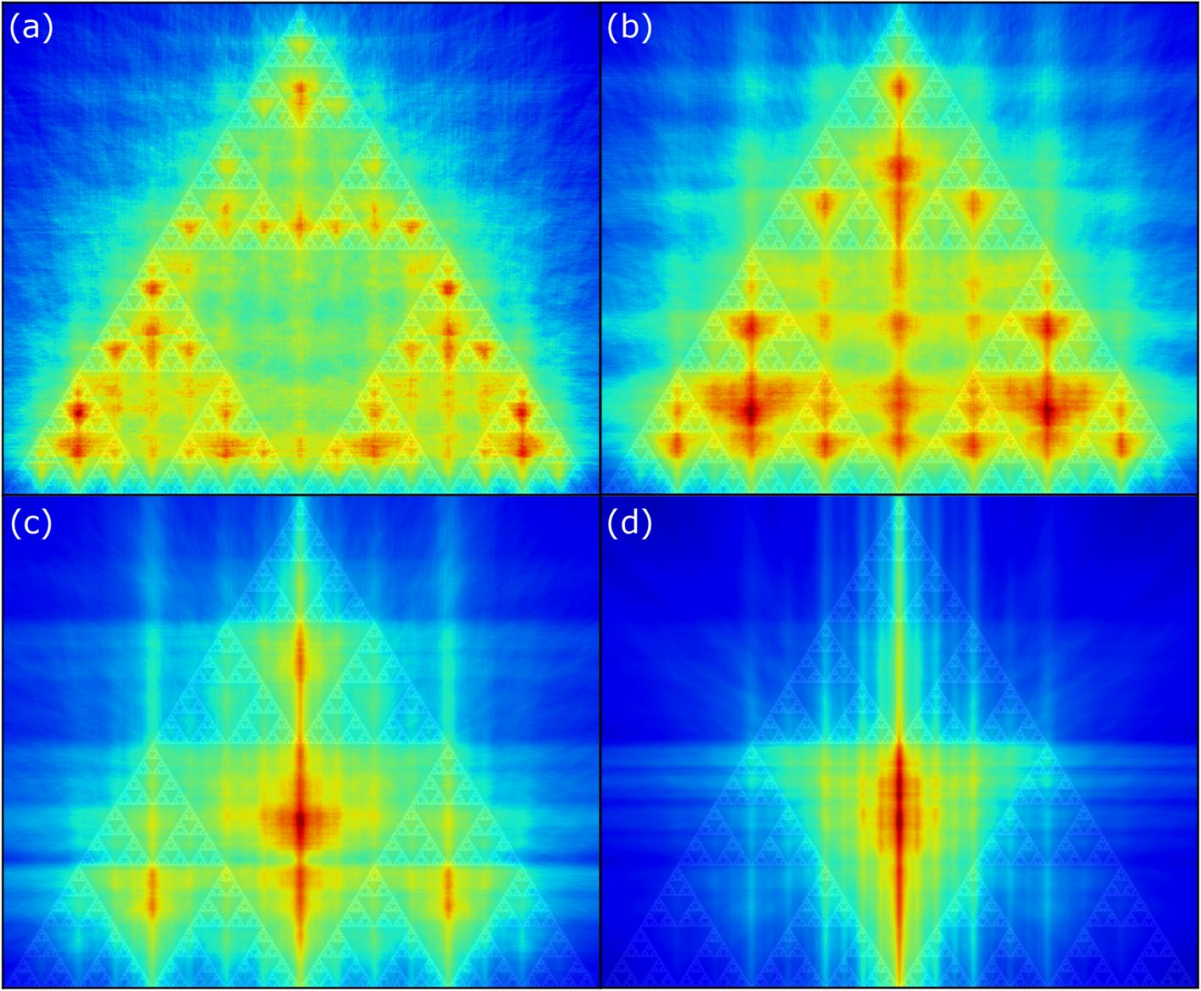
An iterated function system (IFS) fractal of triangles processed through the symmetry density algorithm with distance limits of (**a**) 9%, (**b**) 19%, (**c**) 39%, and (**d**) 78% of the image size, shows that by limiting the definition of coincidence to points of an a priori scale, features at the scale highlighted due to the contribution of equidistant point-pairs across lines of symmetry, while point-pairs off lines of symmetry contributing to noise are reduced.

By eliminating points from the coincidence detection that do not meet some a priori criteria for symmetry, the definition of coincidence is refined and the signal of the line of symmetry will increase. For example, if by a priori knowledge of the image type symmetry greater than some scale can be eliminated, coincidence of pairs of points with distances greater than that scale can be removed from the coincidence detector. A demonstration with a triangular fractal (Fig. 7) shows that by limiting the points available to the coincidence detector lines of symmetry at varying scales are hilighted over the noise generated by the other symmetry scales in the data.

Similarly, when the symmetry is not exact, pairs of points on either side of the quasi-line of symmetry may not contribute to the coincidence detection since they are not precisely equidistant. In this case adding noise to the distance metric will cause at least some of the point-pairs across the quasi-line of symmetry to coincide and contribute to the signal of the quasi-symmetry. Here, instead of eliminating point-pairs that by a priori knowledge only contribute to noise, the definition of coincidence is relaxed to allow approximate lines of symmetry to become visible at the expense of noise.

**Figure 8:**
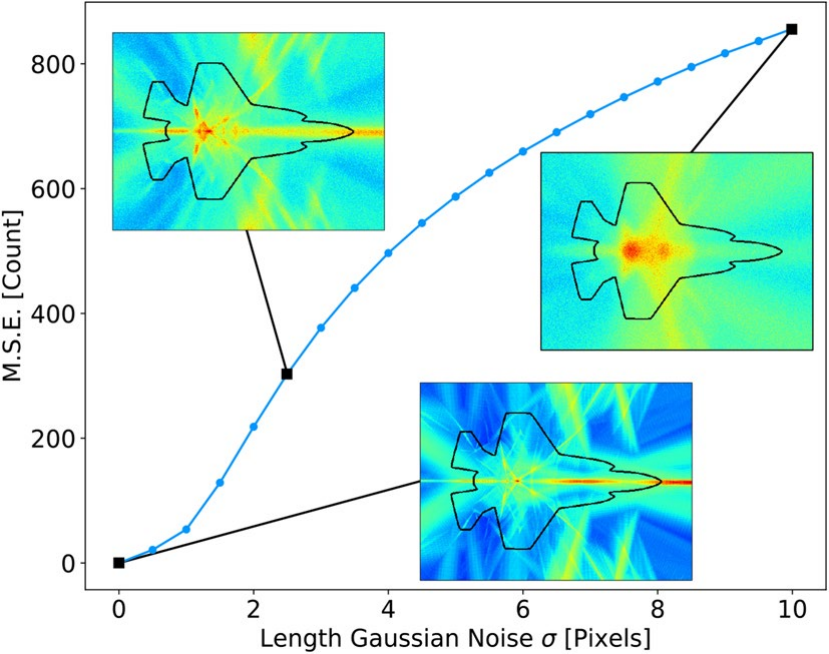
Guassian noise added to each measure of distance increases the mean squared error (MSE) when compared to the zero noise case. For noise with standard deviation less than or equal to the bin size (in this case 1), output MSE is only slightly impacted with a maximum MSE of 53. For noise with standard deviation greater than the bin size, MSE increases steeply before leveling as the noise standard deviation reaches ten times the bin size, and the relative change in the output density with increasing noise power is reduced.

To evaluate the effect of noise on the network, we simulated the outline of the F-35 with Gaussian noise added to the length delay with a standard deviation ranging from 1 to 5 (Fig. 8). The results show that small features with narrow bands of coincidence are quickly eliminated while broad ranging symmetry points from across the network increase in dominance.

This can be explained by the strict coincidence detection of the network. When two points are pushed away from each other even by a distance of one pixel, the coincidence detection will not happen. In the case of the thin line of symmetry between equidistant lines, for example near the nose of the aircraft, adding noise disperses the boundary between them. Alternatively, in cases where the symmetry is not exact, for example near the tail of the aircraft, adding noise creates new coincidence points as some previously unaligned spikes are now brought into alignment with each other. This demonstrates the value of adding noise when considering shapes with less strictly defined symmetric features.

## Discussion

In conclusion, we have presented a novel algorithm for finding a scalar field representing the symmetry of points in a multi-dimensional space. We have demonstrated that time synchronization in the input values of spiking neural networks, with the appropriate choice of threshold and spike period, results in the identification of output neurons along points of high symmetry density to the network inputs. We have demonstrated an implementation of the symmetry selective LIF neural network in common hardware with a high speed, 2.8 MHz identification of symmetry points in an $8\times 8$ Manhattan metric space. Our results confirm that utilizing only the delay and coincidence detecting properties of a single layer of neurons in a spiking neural networks naturally leads to effective symmetry identification. A greater understanding of symmetry perception in artificial intelligence will lead to systems with more effective pattern visualization, compression, and goal setting processes. Future research should focus on optical implementations of the presented findings a) to harness the information parallelism of bosonic photons, b) to capitalize on the high energy efficiency of photonic and nanophotonic optoelectronics which only require the micrometer-small active devices to the electrically addressed enabling 100’s of atto-Joule efficient active optoelectronic devices, and (c) to enable high-data throughput links and platforms^34–43^.

## Methods

### Python implementation

We begin with a Python simulation using a sparse matrix representation of input nodes. For each element of the output mesh, the distance to every input node is calculated using Euclidean distance and rounded to into a configurable binning decimal place. The result is stored in a list. The most frequent distance in the list is found and the count of the most frequent distance is placed in the output mesh.

**Figure 9:**
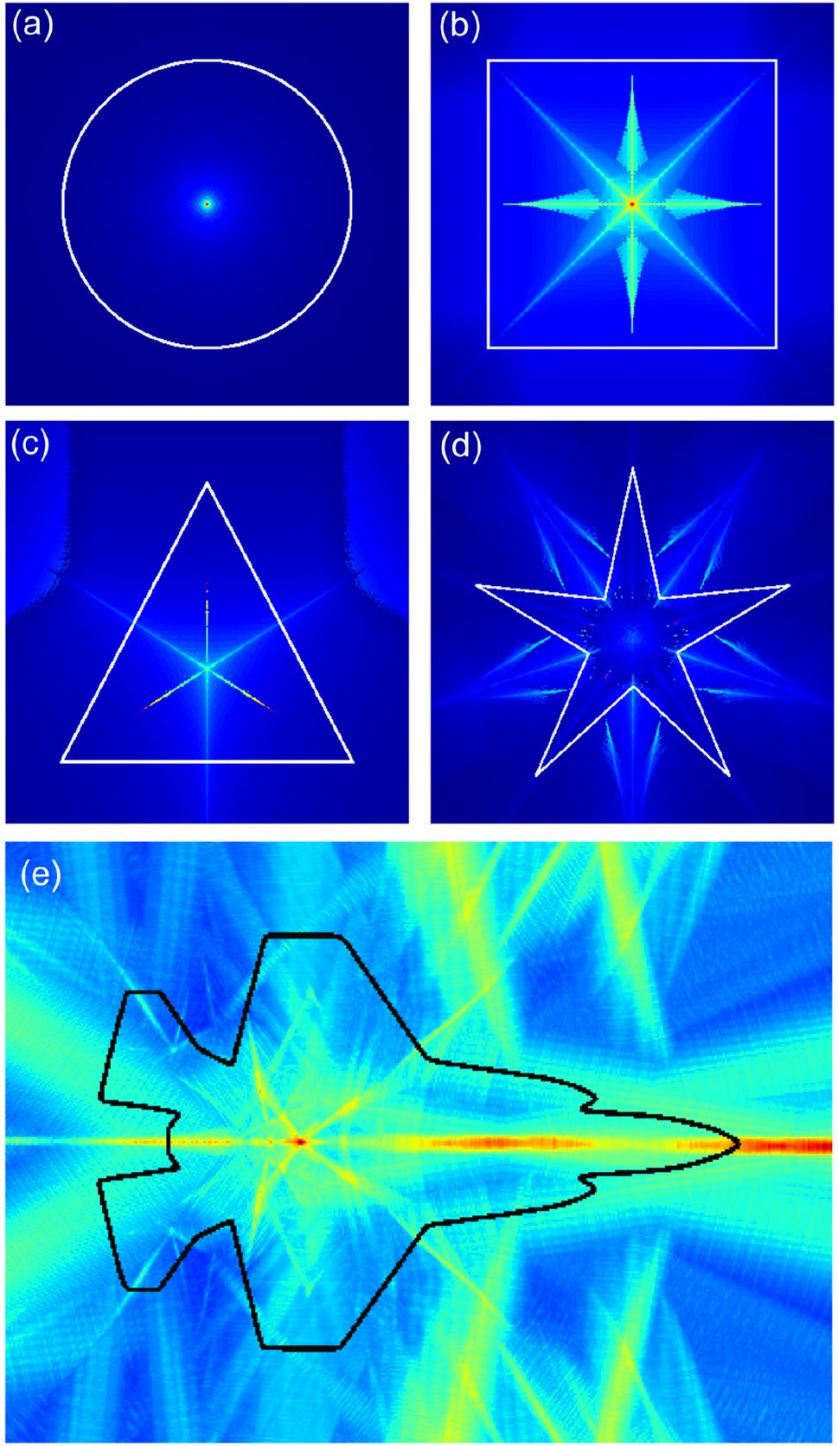
The result of an implementation of Algorithm 1 in Python for a circle (**a**), square (**b**), triangle (**c**), star (**d**), and an aircraft shows a point and fractal like mirroring of the mirror symmetry density function.

Using this implementation we calculate the symmetry space for input data from low to high complexity (Fig. 9).

### Implementing LIF with digital logic

Each neuron is represented as a leaky accumulator. At each clock cycle all of the neuron inputs are added to a value stored in an accumulation register, the result of the summation minus the leak value is then stored back in the accumulation register. If the accumulation register surpasses the value of a fixed threshold, or, alternatively as a configurable threshold stored in a threshold register, the accumulator is reset to zero and a value appears at the neuron’s output. If the accumulation value does not surpass the threshold, zero appears at the neuron’s output.

Neuron-to-neuron connection delay is represented either as queue, where the outputs are placed into a First-In-First-Out (FIFO) queue, or as a pulse value and countdown register. When delay is represented as a queue, at each clock cycle a single value is added from the output neuron to each output connection’s FIFO and a single output is removed from each FIFO at the output neuron. The length of each FIFO is proportional to the delay being represented. When delay is represented as a countdown register, at each clock cycle if the neuron’s output value is positive a countdown register is initialized with a count proportional to the represented connection delay and a value of the neuron’s output value. At each clock cycle each countdown register is decremented. When a countdown register reaches zero, its pulse value register is placed at the input of the output neuron.

### FPGA implementation

To demonstrate spatial symmetry recognition via coincidence detection of LIF networks on actual hardware, we implemented a simple LIF spiking neural network on a Xilinx Zynq™ FPGA. Our LIF neural network consists of an $8\times 8$ input array connected to an $8\times 8$ neuron output array. Each output neuron is connected to every input by a shift register of length proportional to the Manhattan distance from the point (Ox,Oy) at the output to the point (Ix, Iy) at the corresponding input. This results in 4096 shift registers with a maximum length of 16. Each output neuron consisted of a two-stage adder followed by an accumulation register. Each adder includes a configurable constant leaky term that subtracted the configured leak from the accumulation register at each time interval. Each accumulator is connected to a threshold level. If the accumulator passes the threshold, a second single-bit register is set to 1 to indicate firing of the output neuron. The latency from input to output in this implementation is proportional to the length of the longest shift register, 16 in this case, plus the accumulation time, 2 in this case, for a total of 18 clock cycles. The network is clocked at a constant clock speed of 50 MHz for approximately 2.8 MHz of $8\times 8$ symmetry operations. The output of the symmetry LIF neural network was recorded over time for the elementary case of a line between two points. Our results confirm that spiking LIF neural networks indeed act as symmetry detectors, highlighting equidistant points in spatial data.

### Graphical processing unit (GPU) implementation

Spiking neural network hardware is not widely available today. Instead GPUs dominate image and video processing. To demonstrate the symmetry density efficiently, we created a simplified variation of the symmetry density algorithm tailored for the GPU (Algorithm 2).

Instead of creating a histogram of distances at each point in the output, the simplified algorithm attempts to create a density space of bisection lines. It begins by selecting all points of the input that are greater than some threshold and placing them in the 1D array P. It then loops every pair of points in P. It selects the line of points that bisect the point pair using a modified version of Bresenham’s line algorithm^44^ where the slope is inverted to draw a line bisecting the two points rather than a line between the two points.

As with many parallel processors, performance bottlenecks occur in the GPU when many cores need to operate on the same memory space. In this case critical sections are protected by multiple simultaneous operations by mutexes. In the case of Algorithm 2 there are two potential critical sections. The first is in the building the set of points above threshold (Algorithm 2 Line 6). The second is in incrementing the output array (Algorithm 2 Line 9). With these two critical sections the parallel performance of the algorithm is limited. 
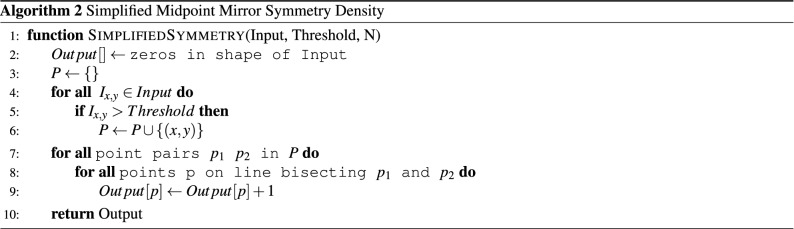


To eliminate the critical sections and improve performance, a heuristic approach may be taken (Algorithm 3). Here the first critical section is removed by randomly indexing into a fixed length threshold point array P placing the selected points at multiple places in P, K times. This creates a random space that contains the threshold points in a random order, eliminating the need to access P within a critical section. 
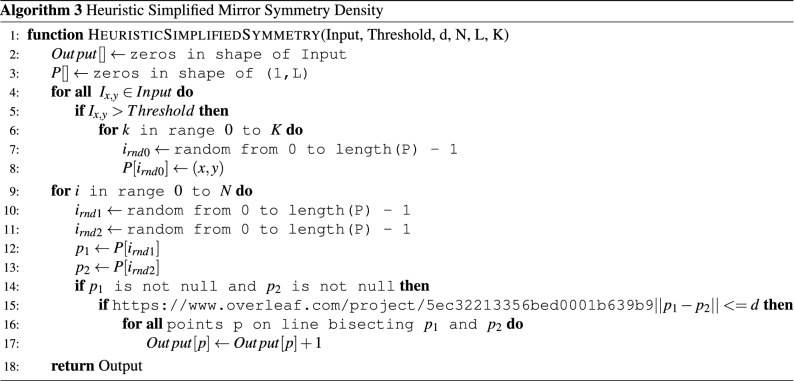


The second critical section is removed by randomly selecting two points from P to calculate the midpoint. Since the midpoint is randomized, the probability of two cores selecting the same index of Output goes to zero as the number of points in P becomes much greater than the number of cores. Further, if two cores do conflict the value is either not incremented, or incremented twice. For large values of N, this simply adds a small amount of noise to the resulting output.

In this way, the heuristic version of the symmetry density algorithm eliminates all critical sections, allowing both for loops to execute across any number of cores simultaneously, at the cost of adding a small amount of noise to the output image.
